# Perlecan controls neurogenesis in the developing telencephalon

**DOI:** 10.1186/1471-213X-7-29

**Published:** 2007-04-05

**Authors:** Amparo Girós, Javier Morante, Cristina Gil-Sanz, Alfonso Fairén, Mercedes Costell

**Affiliations:** 1 Department of Biochemistry and Molecular Biology, Universitat de València, Av. Dr. Moliner 50, 46100 Burjassot, Spain; 2 Instituto de Neurociencias de Alicante, CSIC, Universidad Miguel Hernández, 03550 San Juan de Alicante, Spain; 3 Center for Developmental Genetics, Dept. Biology, New York University, New York, NY 10003, USA

## Abstract

**Background:**

Perlecan is a proteoglycan expressed in the basal lamina of the neuroepithelium during development. Perlecan absence does not impair basal lamina assembly, although in the 55% of the mutants early disruptions of this lamina conducts to exencephaly, impairing brain development. The rest of perlecan-null brains complete its prenatal development, maintain basal lamina continuity interrupted by some isolated ectopias, and are microcephalic. Microcephaly consists of thinner cerebral walls and underdeveloped ganglionic eminences. We have studied the mechanisms that generate brain atrophy in telencephalic areas where basal lamina is intact.

**Results:**

Brain atrophy in the absence of perlecan started in the ventral forebrain and extended to lateral and dorsal parts of the cortex in the following stages. First, the subpallial forebrain developed poorly in early perlecan-null embryos, because of a reduced cell proliferation: the number of cells in mitosis decreased since the early stages of development. This reduction resulted in a decreased tangential migration of interneurons to the cerebral cortex. Concomitant with the early hypoplasia observed in the medial ganglionic eminences, Sonic Hedgehog signal decreased in the perlecan-null floor plate basal lamina at E12.5. Second, neurogenesis in the pallial neuroepithelium was affected in perlecan deficient embryos. We found reductions of nearly 50% in the number of cells exiting the cell cycle at E12–E13. The labeling index, which was normal at this age, significantly decreased with advancing corticogenesis. Moreover, nestin^+ ^or PCNA^+ ^progenitors increased since E14.5, reaching up to about 150% of the proportion of PCNA^+ ^cells in the wild-type at E17.5. Thus, labeling index reduction together with increased progenitor population, suggests that atrophy is the result of altered cell cycle progression in the cortical progenitors. Accordingly, less neurons populated the cortical plate and subplate of perlecan-null neocortex, as seen with the neuronal markers β-tubulin and Tbr1.

**Conclusion:**

As a component of the basal lamina, perlecan both maintains this structure and controls the response of the neuroepithelium to growth factors. Less mitotic cells in the early medial ganglionic eminences, and impaired cell cycle progression in the late neocortex, suggests insufficient recruitment and signaling by neurogenic morphogens, such as SHH or FGF2.

## Background

During the histogenesis of the cerebral cortex, neural precursor cells in the anlage of the cortical hemispheres as well as in the subpallium withdraw from the cell cycle and migrate in an ordered manner following multidirectional pathways to generate a functional neuronal architecture [1]. Proliferation and cell fate determination in the developing brain are extrinsically regulated by multiple interactions among a large number of secreted molecules, such as Sonic Hedgehog (SHH), epidermal growth factor (EGF), and fibroblast growth factors (FGFs) [2,3], which usually act in a concentration-dependent manner. The concentrations of these morphogens are modulated in turn by components of the extracellular matrix (ECM).

Perlecan is one of the most ubiquitous and multifunctional ECM proteins. The proteoglycan is expressed during the prenatal stages of brain development in the basal laminae of the neuroepithelium and of blood vessels [4]. Perlecan binds with varying avidity to many diverse macromolecules. These include cell-surface receptors such as β1 and β3 integrins [5,6] and α-dystroglycan [7,8]; other ECM proteins such as nidogen, collagen IV, laminin, fibulin and fibronectin [9-11]; a number of signaling molecules such as FGF2 [12], FGF7 [13], platelet-derived growth factor B (PDGF-B) [14] and Sonic Hedgehog (SHH) [15]; and enzymes such as acetyl cholinesterase [16]. In particular, it is well known that the heparan sulfate (HS) moieties of perlecan interact with growth factors, regulating their interaction with cell surface receptors [17].

Analysis of perlecan-null mice has demonstrated essential roles of this proteoglycan during development [4,18,19]. The absence of perlecan does not compromise assembly of basement membranes. However, perlecan-deficient embryos showed severe chondrodysplasia, life-threatening malformations of the heart outflow tract, as well as impaired telencephalic development. Perlecan-null mice die before birth due to their bone and heart malformations.

In a previous publication, we reported that many of homozygote perlecan-null embryos presented exencephaly [4]. We showed that exencephaly was not due to impaired neural tube closure, but to an ulterior disruption of the developing cerebral cortex caused by the invasion of neural cells into the meningeal layers [4]. Interestingly, even perlecan-null embryos without exencephaly showed manifold morphogenetic alterations of the brain. This observation suggested that these embryos might serve to analyze major physiological functions of the protein in telencephalon morphogenesis. In the present investigation, we studied in detail brain development in perlecan-null embryos that do not show exencephaly. We conclude that perlecan potentiates cell cycle progression and neuronal differentiation in the cerebral hemispheres and ventral forebrain. Our data suggest that perlecan could critically regulate the availability of a crucial morphogen, such as SHH, in the floor plate.

## Results

### Two brain phenotypes converge in perlecan-null embryos

In the present analysis, we selected perlecan-deficient embryos that were not exencephalic. Whilst all homozygote perlecan-null embryos depicted cerebral ectopias, only about the 55% developed exencephaly. Ectopias are invasions of the meningeal layers by neuroblasts after the disruption of basal lamina. As previously reported, in the most early (about E10.5) and severe cases these invasions opened the neural tube in the frontal part of the cerebral hemispheres [4] driving to exencephaly, which prevents the brain to complete its development. Milder ectopias did not induce *de novo *generation of cortical openings. In these cases, ectopic cells did not proliferate and remained trapped within the marginal zone generating distortion of the cortical layers but allowing the brain to complete development. Non-exencephalic embryos, which represent the remaining 45% of the perlecan-deficient embryos, invariably displayed a hypoplasic brain phenotype. Typically, in these animals we observed the cerebral cortex to proceed along development. It showed an obvious lamination, with a clear differentiation of cortical compartments such as preplate and, later, marginal zone, subplate and the cortical plate. Occasionally, and most commonly near the rostral pole of the telencephalon, isolated ectopias were evident. We considered these brains useful to analyze critical functions of perlecan during corticogenesis. As a control in assessing such a hypoplasic phenotype, after a visual inspection of the embryos, we sectioned the brains serially and observed all the sections carefully. We discarded as exencephalic those brains showing openings of the cerebral wall. For the current investigation, we have analyzed 24 perlecan-null non-exencephalic embryos and 36 wild-type ones, ranging from embryonic day (E) 10.5 to E17.5, distributed in 18 litters.

### Non-exencephalic perlecan-deficient embryos are microcephalic

**Figure 1:**
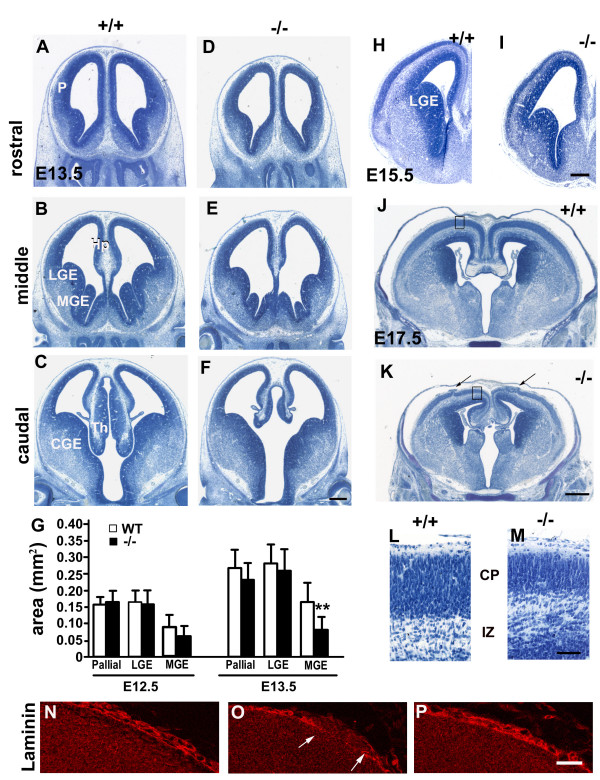
**Microcephaly in perlecan-null embryos**. (A-F) Series of Nissl stained coronal sections of E13.5 forebrain at rostral (A, D), middle (B, E) and caudal (C, F) levels. The brains of a wild-type (A-C) and a perlecan-null (D-F) embryos are shown. Note a reduction in the size of the ventral forebrain in the perlecan-null brains, particularly affecting the medial structures, such as MGE, hippocampus, and rostral part of the thalamus. (G) Quantification of pallial, MGE and LGE cross-sectional area per hemisphere in sections at the middle level. Results reveal that whole brain size is reduced variably in perlecan-null embryos, but that at E13.5 all perlecan-null embryos have severely underdeveloped MGE as compared with their wild-type littermates. Two litters at E12.5 and three litters at E13.5 were used; n = 2 embryos at E12.5 and n = 6 embryos at E13.5. ** *p *< 0.01. (H, I) Nissl stained coronal sections at E15.5 in wild-type (H) and perlecan-null (I) embryos. Ventral forebrain underdevelopment extends significantly to LGE and paleocortex. Note the reduction of cortical plate thickness in the perlecan-null cortex. (J-M) E17.5 wild-type (J, L) and perlecan-null (K, M) embryos. Panels L and M represent enlargements of the regions boxed in J and K, respectively. The ventral forebrain is reduced in size in the perlecan-null embryo, and the cortical plate is thinner. Additionally, in the perlecan-null telencephalon, the onset of neuronal ectopias (arrows in K) is frequent in the most antero-dorsal region of the cerebral hemispheres. (N-P) Antibodies to laminin highlight the meningeal basal lamina, the meninges and the blood vessels in the wild-type (N) and perlecan-null (O and P) embryos. In the perlecan-null, disruption of the basal lamina is observed in an ectopic area (indicated by arrows in O). However, in the rest of the basal surface of the perlecan-null brain, laminin deposition is continuous (P). Scale bars: 250 μm (A-F, H-I), 500 μm (J, K), 50 μm (L, M), 40 μm (N-P). Abbreviations: CGE, caudal ganglionic eminence; CP, cortical plate; Hp, hippocampus; IZ, intermediate zone LGE, lateral ganglionic eminence; MGE, medial ganglionic eminence; P, pallium; Th, thalamus.

The non-exencephalic perlecan-null embryos showed drastic reductions in the size of telencephalon. It is important to recall that perlecan-null embryos do not display reductions in the size of the rest of the body [4]. Fig. 1 shows coronal sections of E13.5 wild-type (A-C) and perlecan-null (D-F) telencephalon at different anteroposterior levels. Figure 1G illustrates the comparison of the cross-sectional areas of the cortical primordium (Ctx), lateral ganglionic eminence (LGE) and medial ganglionic eminence (MGE) measured in sections similar to those in Figure 1B and 1E. At E12.5 and E13.5, the overall morphology was normal in the mutant telencephalon. Although there were pallial reductions in some cases, a constant underdevelopment was evident in the MGE and the rostral part of hippocampus primordium (Hp). In all perlecan-null embryos, the MGEs cross-sectional area ranged between 24% and 70% of that of wild-type embryos of the same litters. At E15.5, LGE in addition to the MGE (Fig. 1H – I) and the CGE (not shown) showed striking reductions in size as compared with wild-type littermates. Moreover, at this stage, neocortex and paleocortex primordia were markedly thinner.

In the E17.5 perlecan-null embryos (Fig. 1J – M), the cross-sectional area of the brain was reduced to about 80% of that in their wild-type littermates. At this age, brain reduction resulted mainly from a decrease in the cross-sectional area of the ventral telencephalon, but also the cerebral wall was significantly thinner mainly due to reduced cortical plate thickness (Fig. 1L – M). In addition, a number of ectopias were visible in the cortical plate, which distorted the cortical laminar pattern (arrows in Fig. 1K). The ectopias consisted of groups of early-generated neurons that invaded the meningeal layers disrupting the basal lamina, as shown by laminin immunostaining (Fig. 1N – O). This figure shows that in both perlecan-null embryos and wild-type littermates, basal lamina assembled normally in the cerebral surface (Fig. 1P).

In conclusion, the perlecan-null embryos that complete their prenatal development were microcephalic. The cerebral hypoplasia progressed following a ventromedial-to-laterodorsal sequence along brain development. The brains maintained basal lamina continuity, interrupted by some isolated ectopias.

### The cell proliferation in the forebrain decays in the absence of perlecan

**Figure 2:**
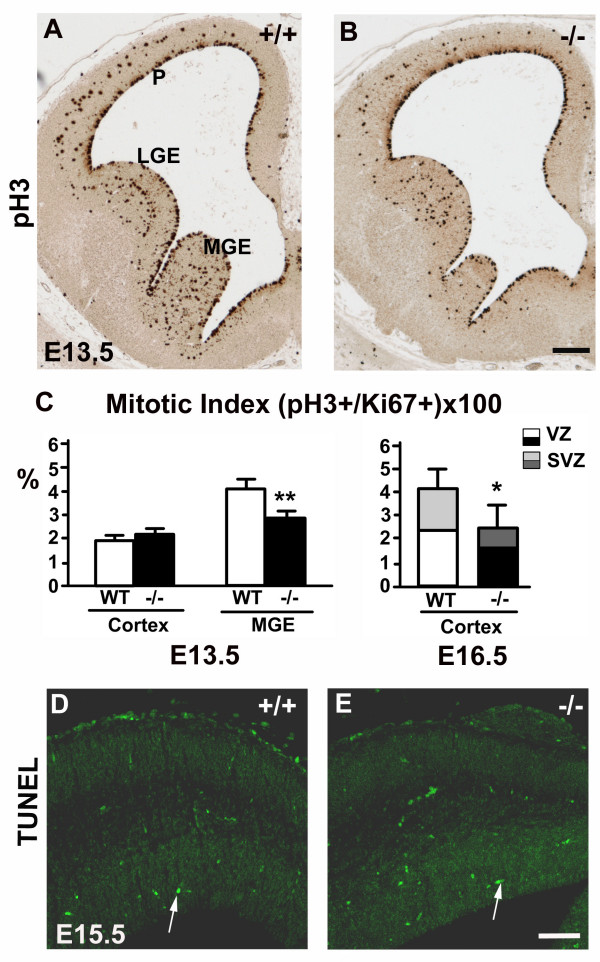
**Cell proliferation decays in the absence of perlecan**. (A, B) Mitotic cells in M phase immunodetected with an anti-phospho-histone H3 antibody in the telencephalic vesicles of E13.5 wild-type (A) and perlecan-null (B) embryos. The neocortex shows two well-differentiated mitotic populations: the ventricular primary population in the VZ, and the basal progenitors in the SVZ that emerges in the middle of the pallium at E13.5. (C) The mitotic index (the percentage of phospho-histone H3-labeled cells among Ki67^+ ^progenitors) shows significant differences in the MGE, but not in the neocortex at E13.5. At E16.5, the mitotic index in the neocortical primordium is reduced to about a 60% of that in wild-type embryos, affecting both the VZ and the SVZ mitotic populations. Values are the mean ± SEM, n = 5 at E13.5; n = 4 at E16.5. * *p *< 0.05; ** *p *< 0.001. (D, E) TUNEL staining of E15.5 wild-type (*D*) and perlecan-null (*E*) dorsal neocortex. As in the wild-type littermates, very few TUNEL-positive cells (arrows in D and E) were observed in perlecan-null embryos. Scale bars: 150 μm (A, B), 80 μm (D, E).

To understand the cellular mechanisms involved in the brain hypoplasia, we measured proliferation in the ventricular (VZ) and subventricular zones (SVZ) of the pallium and subpallium. To this purpose, we first used an antibody to the M phase marker histone H3 phosphorylated on Ser 10 [20] (Fig. 2A – B). We quantified the mitotic index as the ratio between the number of metaphase phospho-histone H3^+ ^(pH3^+^) cells referred to the number of progenitor cells in the same area, marked with antibodies to Ki67 (Fig. 2C; see Methods). We found that cells in metaphase distributed uniformly along the ventricular surface of the telencephalic vesicles until E12.5 (data not shown). At E13.5 (Fig. 2A – B), a secondary mitotic population, called basal progenitors, appeared in the SVZ of the cortical primordium. In parallel with the progression of hypoplasia, the mitotic index was reduced in the perlecan mutants, starting in the medio-ventral telencephalon and reaching the cortex at advanced corticogenesis. At E13.5, the perlecan-null brains had a mitotic index similar to that of wild-type embryos in the cortex, but displayed a reduced index in the MGE (2.6% in perlecan-null mice vs. 4.0% in wild-type mice; Fig 2C). At E16.5, the mitotic index was reduced in the perlecan-null neocortex to about a 60% of that in the wild-type, and the reduction affected both the VZ and the SVZ (basal) progenitors (Fig. 2C). Thus, reduced proliferation seemed to contribute to brain hypoplasia.

Together with the decay in the mitotic index, an augmented rate of programmed cell death may also account for the reduced brain size. We examined the telencephalon for the presence of DNA fragments that are direct evidence of programmed cell death using TUNEL staining. No significant changes in the number of TUNEL^+ ^cells were detectable in the neocortex and basal ganglia of perlecan-null embryos. In Fig. 2D,E we show the TUNEL stained neocortex of E15.5 wild-type and perlecan-null embryos. At this age, few apoptotic cells were detectable by TUNEL staining in the developing cortex, and we observed no significant differences in the perlecan-null embryos. Thus, cell death must not be a major agent in generating underdeveloped cerebral walls and subpallium in perlecan mutants. However, the contribution of cell death to brain hypoplasia cannot be discarded completely, since it is known that the TUNEL method does not detect all cells that die during prenatal development [21].

### In the perlecan-null neocortex, the labeling index is normal at the beginning of cortical neurogenesis, but decreases at late stages

The impaired mitotic index observed in the pallium suggests that low proliferation of cortical progenitors in the VZ and SVZ might contribute to the reduction of cortical size. The onset of such a process of low proliferation in the cerebral wall occurred at the time corticogenesis is well advanced, i.e., later than in the ventral forebrain. We studied in detail proliferation and neuronal differentiation in the cortex at two representative time points: E12.5–E13.5, which represents an early stage of corticogenesis, and E16.5, which represents a late stage.

**Figure 3:**
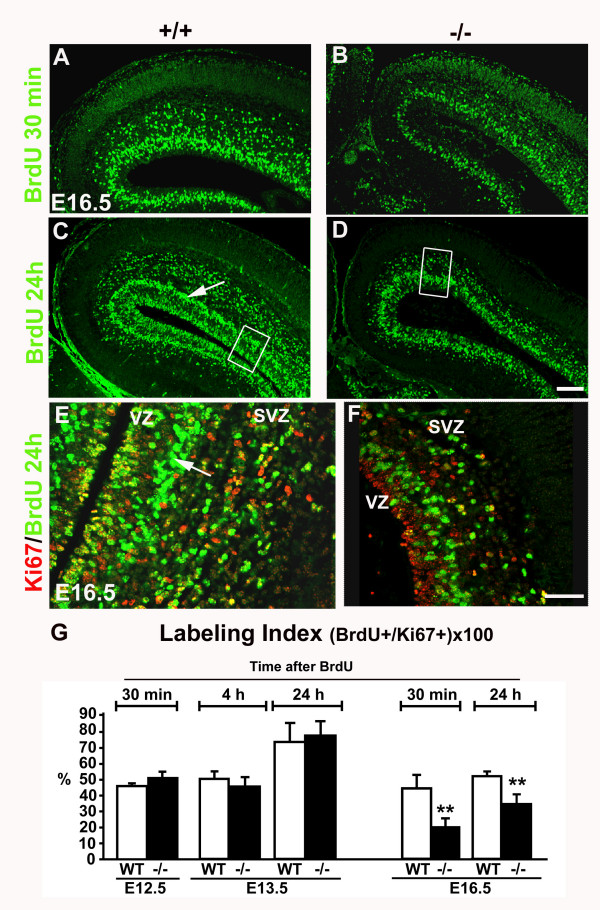
**The labeling index is normal at the beginning of cortical neurogenesis, but decreases at late stages in the perlecan-null neocortex**. (A-D) Immunofluorescence for BrdU (green) in the cortical primordium after a 30 min (A, B) and a 24 hours (C, D) survival to a BrdU pulse in wild-type (A, C) and perlecan-null (B, D) E16.5 embryos. In both cases, less BrdU^+ ^cells are seen in the perlecan mutants. (E-F) Double immunofluorescence for BrdU (green) and Ki67 (red) in the pallium of E16.5 wild-type and perlecan-null embryos after a 24 h survival to a BrdU pulse at E15.5. Note the panels are high magnification views of the boxed areas in C, D. Observe abundant double-labeled cells (yellow) in the VZ and a thick layer containing BrdU^+ ^cells (and no Ki67^+ ^cells) in the SVZ of wild-type embryos, corresponding to the newly generated neurons (arrow in C and E). Observe the reduced BrdU incorporation in the perlecan-null dorsal cortex, affecting both the VZ and the SVZ. (G) Labeling index (the percentage of BrdU^+ ^cells among Ki67^+ ^progenitors) in cortical sections after 30 min, 4 hours and 24 hours survival to a BrdU pulse at E12.5, E13.5 or E16.5. Means ± SEM values are shown. n = 2 embryos for E12.5; n = 4 for E13.5 BrdU 4 h; n = 5 for E13.5 BrdU 24 h; n = 2 for E16.5 BrdU 30 min; and n = 2 for E16.5 BrdU 24 h. ** *p *< 0.001. No significant changes of labeling index are evident in the perlecan-deficient embryos at E12.5 and E13.5, but at E16.5 the index is significantly reduced. Scale bars: 100 μm (A-D), 40 μm (E, F).

To analyze the rates of proliferation of neuronal precursors in the cortical primordium, we used BrdU pulse labeling and survival periods of either 30 min, 4, or 24 hours combined with Ki67 staining (Fig. 3). Thirty min after a BrdU pulse, BrdU-labeled cells appeared distributed throughout the VZ and SVZ (Fig. 3A – B). At E13.5 there was no change in the distribution and quantity of BrdU-labeled cells (data not shown). However, as shown in Fig. 3A and 3B, at E16.5 both VZ and SVZ contained less BrdU^+ ^cells in the perlecan-null embryos than in the wild-type littermates. Twenty four hours after a BrdU pulse (Fig. 3C–F), a dense cell layer appeared between the VZ and SVZ (arrow in Fig. 3C,E). The cells of this layer were BrdU^+ ^but Ki67^- ^and, thus, newborn neurons; they were less abundant in the perlecan-null embryos (Fig. 3D,F).

We calculated the labeling index in non-ectopic areas of the neocortical primordium of perlecan-null embryos, in one litter at E12.5, four litters at E13.5 and two at E16.5 (Fig. 3G), and results were compared to mean values of two wild-type embryos from each litter. Labeling index is the number of BrdU^+ ^cells divided by the number of Ki67^+ ^cells (see Methods).

At E12.5 and E13.5, after different survival times to BrdU pulses, we observed an unaltered labeling index in perlecan-null embryos. However, at E16.5, the labeling index decreased significantly to about 50% in the perlecan-null embryos (Fig. 3G). This was due to both a decrease in the number of BrdU^+ ^cells and to a moderate increase in the size of the progenitor Ki67^+ ^population. In conclusion, the decrease in the labeling index indicates that the cell cycle lengthened abnormally in perlecan-null embryos as corticogenesis proceeds.

### Since the earliest steps of cortical neurogenesis, the fraction of cells exiting the cell cycle decreases in the neocortex of perlecan-null embryos

**Figure 4:**
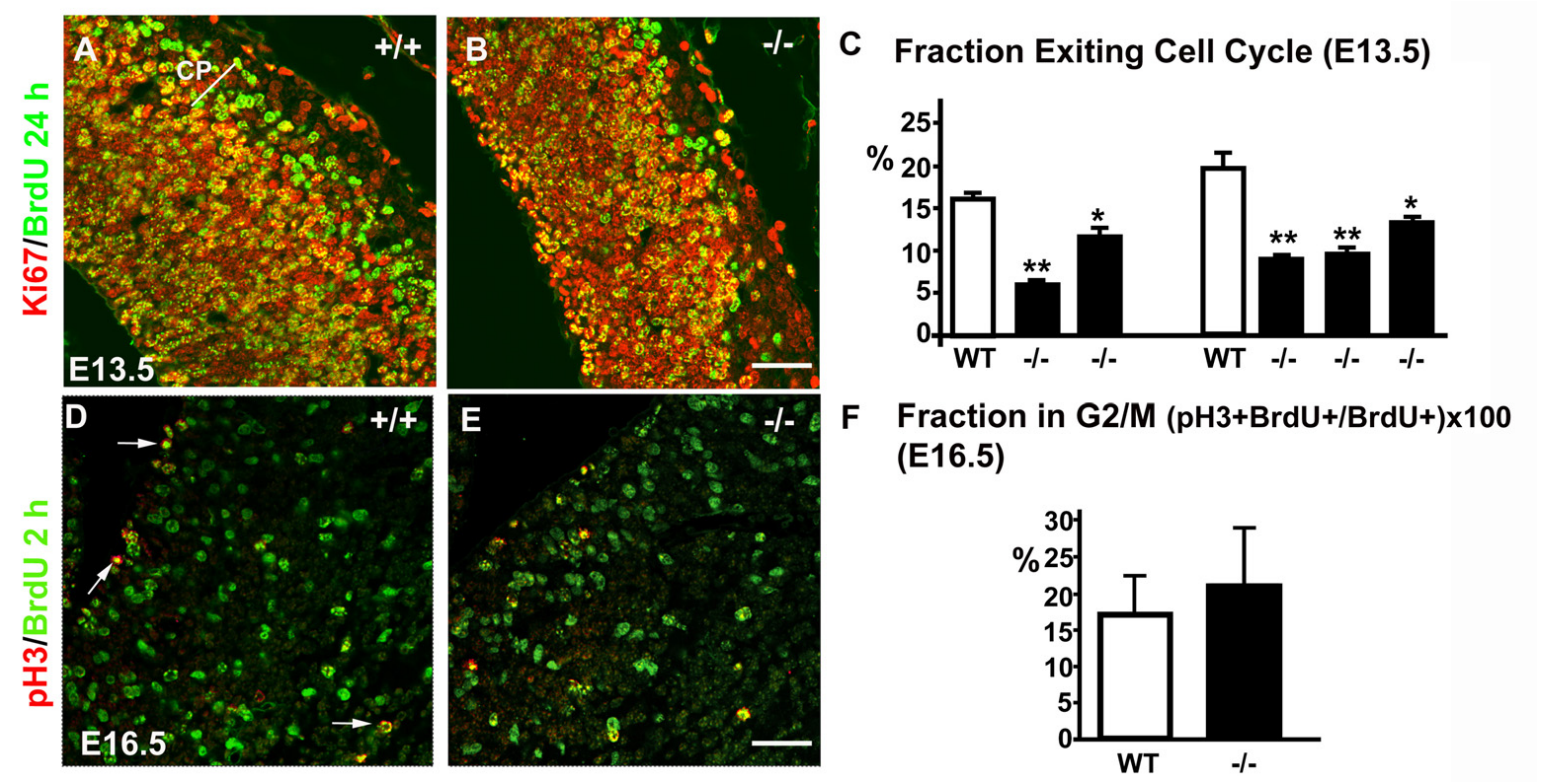
**Defective neurogenesis in the pallium of perlecan-deficient embryos**. (A, B) Double immunofluorescence for Ki67 (red) and BrdU (green) after 24 hours survival to a E12.5 BrdU pulse was used to calculate the fraction of cells exiting the cell cycle (quitting fraction) at E13.5. Cells labeled only with BrdU, a pool no longer dividing, are abundant in the cortical plate in the wild-type embryos (CP in A), but its number in the perlecan-null neocortex decreases considerably (B). (C) Quantification of the fraction of cells leaving the mitotic cycle (quitting fraction: percentage of the number of BrdU^+^, Ki67^- ^cells among the total of BrdU^+ ^cells) in two E13.5 litters. In the perlecan mutants, the number of cells that leave the cell cycle is reduced to approximately a half of that in wild-type littermates, n = 6 slices.* *p *< 0.05 and ** *p *< 0.01. (D, E) Double labeling with phosphorylated histone H3 (red) and BrdU (green) after 2 hours survival to a BrdU pulse, used to compare G_2_/M phase in wild-type (D) and perlecan-null (E) neocortex at E16.5. (F) Quantification of the percentage of cells in G_2_/M cell cycle phase (double labeled cells; arrows in D) referred to the total of BrdU labeled cells. n = 12 slices. Scale bar: 40 μm (A, B, D, E).

To analyze neuronal differentiation, we measured the quitting fraction, i.e., the fraction of cells leaving the cell cycle at a given time point. We selected two E13.5 litters, with two and three perlecan-null embryos each, because cortical neurogenesis is already massive in the wild-type neocortex at this age. We applied a pulse of BrdU and collected the tissue 24 hours later (Fig. 4A – B). We calculated the cell cycle quitting fraction as the proportion of BrdU^+^/Ki67^- ^postmitotic cells among all BrdU^+ ^cells (Fig. 4C). In the perlecan-null embryos, the quitting fraction reached only a 30–70% of the value for wild-type littermates.

Taken together, these results showed that, although the rate of proliferation decayed progressively as corticogenesis proceeds, neuronal differentiation was impaired in the cortical primordium of perlecan-null embryos already at the earliest stages.

The proliferation rate of mammalian cells is generally regulated in the G_1 _phase of the cell cycle. It has been reported that an heparan sulfate-binding growth factor, FGF2, could modulate G_1_/S transition in cerebral cortex [22]. To test whether the absence of perlecan provokes a lengthening of G_1_, we approached the problem indirectly by measuring the length of G_2_. We administered a pulse of BrdU to one dam at E16.5 and collected the tissue 2 hours later; we used such a survival time since it has been established that G_2_/M lasts for about 2 hours at this embryonic age [23,24]. Phosphorylation on Ser10 of histone H3 is required for chromosome condensation during prophase. Using antibodies to pH3, a punctate nuclear labeling occured during late G_2_, to become homogeneous during the M phase (arrows in Fig. 4D). We measured the fraction of cells double-labeled with pH3 and BrdU in the cortical primordium (Fig. 4D – F). In this experiment, nuclei double-labeled with pH3 and BrdU were the nuclei in M phase that were in S phase at the time of the BrdU pulse. We referred the total number of double-labeled cells to the total number of BrdU^+ ^cells in the same section. The proportion was of 16.9 ± 5.3% in two wild-type embryos and of 20.9 ± 7.9% in one perlecan-null embryo (n = 12 sections), but differences were non-significant. Therefore, the length of G_2_/M phase did not change in the perlecan-null embryos at E16.5 and, thus, could not contribute to the detected decay in proliferation rate. Thus, we conclude that the lengthening of the cell cycle, which indicates an impairment of normal cell cycle progression, was mostly due to the lengthening of G_1 _phase.

### Perlecan deficiency alters the size of the neural progenitor population in the ventricular zone

**Figure 5:**
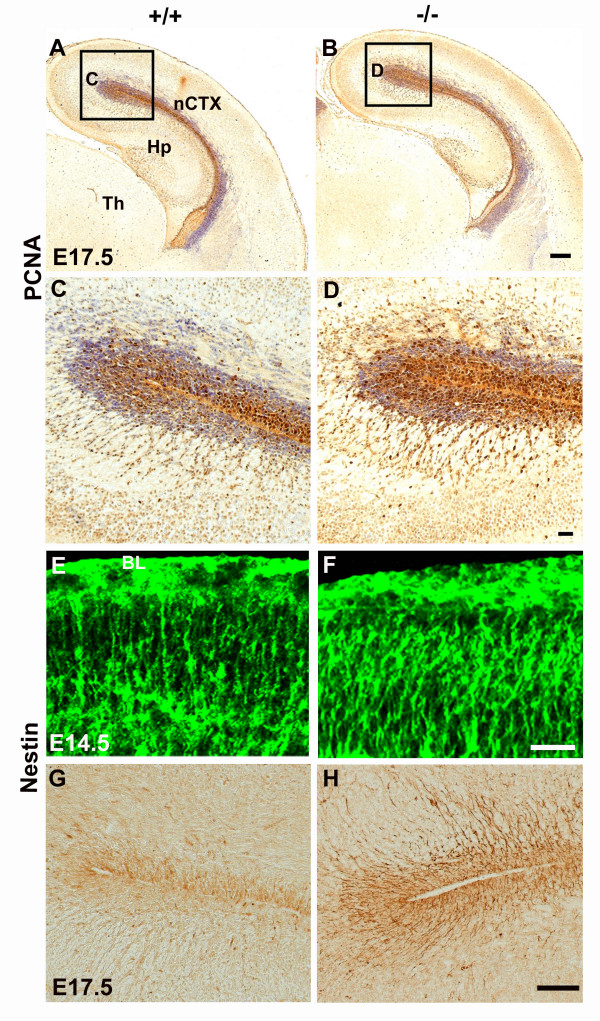
**Perlecan deficiency increases the cortical progenitor pool**. (A-D) Immunostaining for PCNA, a cell cycle marker, in coronal sections of wild-type (A, C) and perlecan-null (B, D) telencephalon at E17.5, Nissl counterstaining. Boxes in (A) and (B) are enlarged in (C) and (D) panels, respectively. (E-H) Nestin expression in coronal sections of wild-type (E, G) and perlecan-null embryos (F, H) at E14.5 (E, F) and E17.5 (G, H). At E14.5, the processes of nestin^+ ^radial glia cells in the marginal zone reach up the pial surface. Note the normal disposition of radial glia endfeet below the basal lamina (BL) in the perlecan-null embryo (F) and in the wild-type (E). Note the marked increase in immunostaining intensity of radial glia in the mutant at both ages. Abbreviations: HIP, hippocampus; nCTX, neocortex; Th, thalamus. Scale bars: 100 μm (A, B), 20 μm (C, D), 30 μm (E, F), 50 μm (G, H).

As described above, we observed an increase in the proportion of progenitor cells labeled by Ki67 antibodies, and that cell proliferation rate diminished, in perlecan-null embryos (Fig. 3E – G). Like Ki67, PCNA is a nuclear protein that is expressed during all phases of the cell cycle [25,26]. We used this antibody to calculate the percentage of cycling cells among the total number of Nissl-stained cells present in the neuroepithelium of the hippocampal and neocortical primordia at E17.5 (Figs. 5A – D). PCNA^+ ^cells represented a 4 ± 0.9%, in wild-type embryos and a 6 ± 0.5% in the perlecan-null embryos (n = 2 embryos, *p *< 0.01). This difference started at E14.5 (30.7 ± 0.2% vs. 38.9 ± 0.7%; n = 2 embryos, *p *< 0.05), but was non-significant at E12.5–E13.5.

The cortical neuroepithelium has two progenitor populations: radial glia and basal progenitors [27]. Nestin labels radial glia, the progenitor cells in the VZ [28] that extend long processes to the basal lamina. To confirm that perlecan-null embryos had a more abundant progenitor cell pool, we next analyzed nestin expression (Fig. 5E – H) and, in agreement with the results with PCNA and Ki67, nestin labeled the neocortical primordium more intensely in perlecan-null than in wild-type embryos. At E14.5, subpial terminations of nestin^+ ^radial glia in the marginal zone packed more densely in perlecan-null embryos (Fig. 5F), the same as nestin^+ ^radial glia in the VZ at E17.5 (Fig. 5H).

Thus, results of nestin immunostaining were in accord with the abnormally high proportion of VZ progenitor population in the late perlecan null neocortex. It is worth of note that both in perlecan-null and wild-type embryos, radial glia terminations reached the basal lamina, indicating that anchoring of glial endfeet to the pial basal lamina (Fig. 5F) occurred even in the absence of perlecan.

### Different neuronal populations in the cerebral cortex show impaired differentiation in absence of perlecan

**Figure 6:**
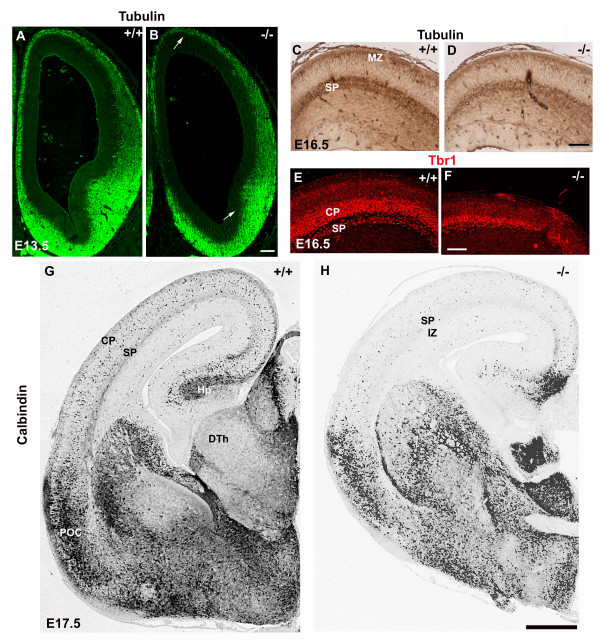
**Less neurons and interneurons are present in the cortical primordium of perlecan-null embryos**. (A-D) β-tubulin type III immunostaining at E13.5 (A, B) and at E16.5 (C, D). At E13.5, the telencephalic vesicles show immunoreactivity in the mantle of the cortical primordium and of the septal and ganglionic eminences. In the perlecan-null, the extension of β-tubulin immunoreactivity is reduced in the pallium and in the subpallium (arrows in B). At E16.5, neocortical β-tubulin expression is strong in the wild-type subplate (C) but clearly reduced in the perlecan mutants (D). (E, F) At E16.5, the transcription factor Tbr1 labels the nuclei of early-born neurons in MZ, CP and SP. In the perlecan-null embryos the number of Tbr1^+ ^neurons decreases severely and the lower tier of the CP is indistinguishable from the SP (F). Note an ectopia at the right hand side of (F). (G, H) Cortical interneurons detected by calbindin immunohistochemistry at E17.5. Comparable rostro-caudal levels are shown. Note in the perlecan-null brain (H) a notorious descent of the packing density of calbindin-immunoreactive interneurons that have invaded the subplate and cortical plate, as well as the hippocampus. Scale bars: 100 μm (A-D), 40 μm (E, F), 200 μm (G, H). Abbreviations: CP, cortical plate; DTh, dorsal thalamus; Hp, hippocampus; IZ, intermediate zone; POC, primary olfactory cortex anlage; SP, subplate.

Next, we confirmed that neuronal differentiation was impaired in perlecan-null cerebral wall. To this end, we used antibodies to β-tubulin type III (Fig. 6A – D) and to Tbr1 (Fig. 6E – F), two neuronal markers. In E13.5 wild-type embryos, β-tubulin marked cells in the mantle of the pallium (i.e. the cortical preplate) and of the septal and ganglionic eminences (Fig. 6A). In the perlecan-null embryos, in accord with the reduced cell cycle exiting population, the thickness of the β-tubulin^+ ^layer was reduced not only in the pallium (see also, Haubst et al., 2006 [29]), but also in the mantle of the septal and ganglionic eminences (Fig. 6B). The decrease of neuronal differentiation in the perlecan-null embryos was even more obvious in the late developing stages. Thus, at E16.5 β-tubulin marked cells within the marginal layer and the cortical subplate (Fig. 6C). As shown in Figure 6D, β-tubulin immunoreactivity in the subplate was significantly less dense in the perlecan-null embryos than in the wild-type littermates. Tbr1 is a T-box transcription factor expressed by diverse populations of early-generated neurons of the developing cerebral cortex [30,31], such as neurons of the marginal zone (MZ), the subplate (SP) and deep cortical plate (CP) (Fig. 6E). In the perlecan-null embryos, the number of Tbr1^+ ^neurons decreased both in the SP and in the deep CP. In addition, Tbr1 labeling revealed that, unlike in wild-type embryos, the subplate did not segregate from the cortical plate in perlecan-null embryos (Fig. 6F). The neurons that populate the MZ and the SP initially reside in the preplate, formed at about E12.5, so that these results suggest that perlecan is critical for the differentiation of the earliest generated neurons at this age. Therefore, the decrease in the neuronal pool in the cortical primordium of perlecan mutants could be due to the impaired rate of differentiation of mitotic cells into postmitotic neurons.

The neocortex is formed mainly by radially migrating neurons born in the pallial VZ and SVZ, but it also receives an important contribution of tangentially migrating interneurons generated in the ganglionic eminences of the basal telencephalon [32] and in other prosencephalic sources [33-35]. To understand how these neuron populations developed in absence of perlecan, we identified a population of presumptive tangentially migrating interneurons by calbindin (CB) immunostaining [32] (Fig. 6G – H). At E17.5, CB^+ ^neurons formed clusters in the basal telencephalon and already distributed within the cortical plate of wild-type brains (Fig. 6G). However, we detected less CB^+ ^neurons in the cortex of perlecan mutants (Fig. 6H) than in the wild-type ones, most likely a consequence of the early hypoplasia observed in the MGE of perlecan-null embryos. Less tangential migration could explain in part the reduction in the cerebral wall thickness.

### Perlecan deficiency alters the distribution of Sonic Hedgehog in the basal forebrain

Perlecan binds signaling molecules that play important roles in forebrain patterning and neurogenesis, such as FGF2 and SHH. FGF2 is a HS binding growth factor involved in brain development. We compared the tissue distribution of this protein in anterior sections of telencephalon of perlecan-null and wild-type littermates between E10.5 and E15.5. However, no changes in the pattern of distribution between wild-type and perlecan-null embryos were evident at any analyzed stage (data not shown).

**Figure 7:**
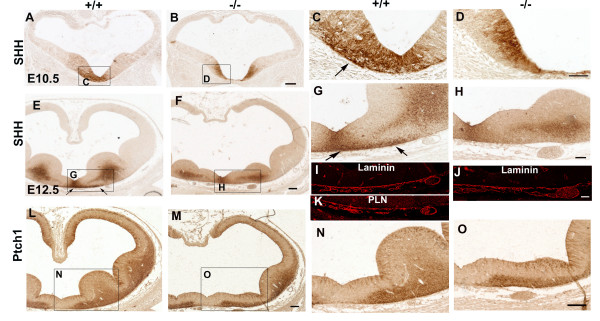
**Distribution of Sonic Hedgehog protein in the telencephalon of perlecan-null embryos**. (A-H) SHH immunostaining in the forebrain of wild-type (A, E) and perlecan-null (B, F) embryos at E10.5 (A-D) and E12.5 (E-H). C, D, G and H are higher magnifications of the boxed areas in A, B, E and F. In the absence of perlecan, the diffusion of SHH into the brain is still present, but there is a significant decrease in the signal intensity in the ventral telencephalon, especially in the medial ganglionic eminences. Note that the floor plate basal lamina shows a strong SHH immunostaining in the wild-type brain (arrows in C and G) whereas no signal is detectable in the perlecan-null embryos (D and H). (I-K) Laminin immunostaining shows basal lamina continuity in the E12.5 wild-type (I) and perlecan-null (J) floor plate. The deposition of perlecan immunostaining in wild-type embryo coincides with that of laminin (K). The region shown in I-K is the same shown between arrows in (G). (L-O) Immunostaining for Patched 1 (Ptch1), the receptor of SHH, in wild-type (L, N) and perlecan-null (M, O) brains at E12.5. Ptch1 distributes in the mantle of the ganglionic eminences and is absent in the midline, the site of strongest SHH signal. In the mutant there is normal distribution of Ptch1. Scale bars: 100 μm (A, B, E, F, L-O), 50 μm (C, D, G, H), 40 μm (I-K).

During early development, the prechordal plate beneath the anterior part of the murine brain produces SHH. From this area, the protein diffuses to the basal part of the forebrain and induces neurons of the ganglionic eminences to initiate their own expression of SHH [36]. At E10.5, we detected the presence of SHH protein in the floor plate and in the medial aspect of the MGE (Fig. 7A, detail in 7C). In the perlecan-null embryos, the territory displaying immunocytochemical signal was reduced (Fig. 7B – D). At E12.5, we detected SHH immunostaining in the floor plate and in the mantle of the MGE (Fig. 7E, detail in 7G), extended caudally across the medial and caudal ganglionic eminences (not shown). In the E12.5 perlecan-null embryos, SHH signal appeared more caudally than in the wild-type (not shown), and the area of SHH signal was reduced, as compared to wild-type littermates (Fig. 7F, detail in 7H). This reduction was particularly evident at the floor plate basal lamina (between arrows in Fig. 7E). Laminin immunostaining shows the continuity of the basal lamina in the floor plate both in wild-type (Fig. 7I) and perlecan-null embryos (Fig 7J). This basal lamina is the place of perlecan deposition (Fig. 7K). These results support the hypothesis that perlecan may play a major role in the regulation of SHH availability in the telencephalon during the early stages of ventral brain development.

We then analyzed the distribution of Patched 1 (Ptch1), the receptor of SHH (Fig. 7L – O). At E12.5, both in wild-type and in perlecan-null embryos, Ptch1 expression did not exactly match SHH signal. In the midline, Ptch1 expression was absent. The LGE showed Ptch1 signal whilst no SHH immunoreactivity was visible in this region. As shown in detail in Fig. 7O, in the perlecan-null embryos the Ptch1 signal in the floor plate was unaltered in places where SHH was nearly absent. However, a less intense Ptch1 immunoreactivity matched the strong decrease of SHH signal in the mantle of the MGE. Thus, in the perlecan mutants at E12.5, Ptch1 expression did not match SHH expression in the floor plate, but it did in the MGEs mantle.

## Discussion

The role of the leptomeningeal basal lamina during brain development remains controversial: whilst some researchers claim that it actively participates in the control of cell proliferation and neuronal differentiation, others only ascribe to it the passive function of maintaining brain structure. In particular, laminin on the one hand and glial integrins and α-dystroglycan on the other are required for anchorage of glial endfeet to the pial basal lamina, and mice defective in these proteins exhibit abundant ectopias and disorganization of the cortical marginal zone [37-40]. In a previous work, we reported that perlecan-null embryos present cortical ectopias or exencephaly. Further, we showed that exencephaly is due to very early disruption of the cortical plate, caused in turn by invasion of neurons into meningeal layers [4]. In the rest of perlecan knockouts, ectopias set off at or before E12.5. With advancing corticogenesis, the clusters of early-generated neurons that form the ectopias settle abnormally and remain trapped in the marginal zone of the cortex.

Among other components of the basal lamina, perlecan bind cell receptors such as integrins and α-dystroglycan [6,8]. However, and in marked contrast with laminin, α-dystroglycan or β1-integrin, perlecan is not essential for extracellular matrix (ECM) assembly [41], and a basal lamina covers most of the cerebral surface of perlecan-null embryos. Nevertheless, the ectopias that form in the perlecan mutants appear to result from defects in the structure of the perlecan-defective basal lamina.

In a recent paper, Gotz and co-workers reported that mutant mice with defects in the continuity of the basal lamina due to a mutated nidogen-binding site in laminin γ1 chain, or to perlecan or α6-integrin knockouts, exhibit normal neurogenesis and proliferation in the ectopic areas where radial glia has lost contact with the basal lamina [29]. These authors infer from these results that the attachment of radial glia endfeet to the basal lamina does not play an important role in the control of radial glia proliferation and fate. This attachment could be, however, functionally relevant for positioning neurons during their radial migration [29]. Based on a much more extended material, we show here that perlecan-null brains undergo cortical neurogenesis indeed, but this process is severely impaired since the earliest stages and implies profound reductions in the thickness of the cortical plate. Moreover, we show here that these alterations affect non-ectopic areas, an aspect unanalyzed in their report. Along these lines, it is also noteworthy that brains of mouse embryos mutated in laminin γ1III4 chain or with a brain-selective deletion of β1-integrin invariably exhibit significant reductions in the size of the telencephalon [38,40]. We consider that this hitherto unexplained defect and our present observations are highly relevant to understand the cues that link basal membrane and neurogenesis.

To ascertain in detail the developmental roles of perlecan and of basal lamina in neurogenesis, we centered our current analysis in brain areas that were devoid of ectopias. In these regions, the basal lamina is present in the brain surface, and radial glia endfeet are in their proper subpial positions.

### Regulation of neurogenesis by perlecan

Atrophy in the perlecan mutants is detectable much earlier in the ventral telencephalon than in the cortical primordium and progresses in a medial-to-lateral direction. Thus, between E12.5 and E13.5, the size of the medial ganglionic eminence in perlecan-null embryos was severely reduced, while those of the LGEs and dorsal telencephalon were normal. During the following days, atrophy extended to the whole ventral telencephalon and paleocortex. A compromised tangential migration of MGE-derived interneurons [42] could perhaps account for paleocortex atrophy. At intermediate stages (E15.5), reduction in size of the ventral telencephalon provided an elongated appearance to the neocortex. Such an elongated aspect has also been described in laminin mutants [29], and invoked to explain the reduction of cortical thickness in these mutants. However, in the late (E17.5) perlecan-null brains the neocortex is not more elongated than in the wild-type, and the whole telencephalon is reduced in size.

During corticogenesis, cell cycle exit, neuronal differentiation and migration are coordinately regulated. At E17.5, the non-ectopic areas of the cerebral cortex of perlecan-null brains had a normal layering thus implying normal radial migration, but the cortical plate was strongly reduced in thickness. The initial defect is a decay of neuronal differentiation: at E13.5, the fraction of cells exiting cell cycle reduced by nearly one-half of normal values in perlecan-null embryos. In good correspondence, immunostaining for Tbr1, which marks different populations of early-generated neurons of the developing cerebral cortex, and βIII-tubulin, a general neuronal marker, revealed a reduction in the packing density of neurons in the perlecan-null cortical plate and subplate. Finally, we found the neocortex of perlecan-null embryos to have much less presumptive interneurons in the cortical plate than their wild-type littermates. The decrease of interneurons is probably the consequence of the initial atrophy that occurs in the ventral forebrain, because the MGE is the major source of tangentially migrating neurons bound to the neocortex [32,43]. In apparent contradiction with the reduced mitotic index, in the perlecan-null primordium we have demonstrated a progressive increase in the proportion of progenitor cells that express nestin or PCNA. At E14.5 the proportion of PCNA^+ ^cells, among the total cell content, increased up to 127%, and at E17.5 reached up to the 150% of the proportion in the wild-type littermates. These data denote an immature status of perlecan defective brain at late corticogenesis.

We note that in other mice mutants, such as the αE-catenin-deficient mice [44] and β-catenin overexpressing mice [45], lack of neuronal differentiation results in bigger brains due to early increases of the progenitor population. This is in contrast to perlecan mutants, where the progenitor population increases only during the most advanced stages of corticogenesis, and the labeling index diminishes concomitantly. This difference might well explain the opposite phenotype observed in αE- and β-catenin *vs*. perlecan mutant mice.

To understand this difference, it is important to stress that in perlecan-null mice the labeling index is unaltered at the onset (E12.5–E13.5) of corticogenesis, but it decreases as corticogenesis proceeds. In mammalian cells, the length of S phase remains relatively constant [46], and no changes in the length of the G_2_/M phases were detectable in perlecan-deficient neocortex. Thus, it seems likely that the lengthening of cell cycle observed in the absence of perlecan is due to an extension of the G_1 _phase. This would in turn result in a progressively increasing fraction of postmitotic cells that cannot become neurons and continue undifferentiated, but have lost the potential to initiate a new S phase. This dormant G_o _state has been described in brain culture, but is considered an abnormal fate option for neuronal precursors in the neuroepithelium that may result from trophic factor deprivation [22]. We will further discuss this aspect below.

Hypoplasia could also result from an increase in the number of dying cells in the perlecan-null brain. We did not detect changes in the apoptotic cell population in the cortex of perlecan mutants at E10.5–11.5 [4] and at E15.5 (present data). Blaschke et al. [21] identified a population of dying cells among the progenitor pool in the developing cortical VZ that accounts an average of approximately 50% of the progenitor population. This population is much larger than the apoptotic pool detected with the standard method of TUNEL or with antibodies to activated caspase 3. Although a detailed study on this apoptotic population in perlecan-null mice is necessary to discard that cell death is playing a role in the cerebral hypoplasia, the observed progressive increase of the progenitor population seems to suggest that apoptosis is not playing a relevant role in brain atrophy.

In summary, less proliferation in both the ventral telencephalon and the cortex will result in decrease of tangentially migrating neurons and less neuronal differentiation in the cerebral cortex, respectively, and may explain the outstanding reduction of cortical thickness that occurs in these knockout mice.

### What are the mechanisms by which perlecan promotes brain maturation?

Diverse morphogens modulate cell division and neuronal fate in a concentration-dependent manner. Perlecan is a large modular proteoglycan that possesses multiple functions associated with both its protein and carbohydrate moieties. In particular, the heparan sulfate chains of the proteoglycan specifically bind two morphogens that are likely candidates to explain the observed brain phenotype, namely Sonic hedgehog (SHH) [15] and fibroblast growth factor-2 (FGF2) [12].

SHH is absolutely required for ventral brain expansion and maturation [2,36,47,48], including the specification of MGE-generated cortical interneurons [49]. In the perlecan deficient embryos, brain atrophy starts at about E12.5 and has a ventromedial-to-lateral progression. SHH protein diffuses into the brain following a ventromedial-to-lateral pattern, which coincides with the direction of brain atrophy progression in perlecan-deficient embryos. The strong immunocytochemical SHH signal found within the floor plate and basal lamina at E10.5–E12.5 probably reflects the fact that SHH is translocated into the brain, where it forms active multimeric complexes [50]. In the perlecan-null embryos, neither SHH distribution within the neuroepithelium nor expression of its receptor, Ptch1, was impaired.

However, a marked reduction in the intensity of the SHH signal in the basal part of forebrain at E10.5–E12.5, and the absence of SHH immunostaining in basal lamina of the perlecan-null embryos suggests a role for the proteoglycan in the spreading of SHH complexes. Accordingly, we propose that perlecan is required to concentrate SHH signal in the floor plate, in order to facilitate its proper delivery to the neural plate at the exact concentration and time needed for correct brain development. Along these lines, the perlecan homologue in *Drosophila*, *trol*, regulates the timing of neuroblast proliferation by modulating FGF and Hedgehog signals [15]. Further, SHH interactions with heparan sulfate proteoglycans promote maximal proliferation of postnatal granule cells [51]. In addition to its role in ventral neural tube patterning, SHH participates in the control of progenitor cell number in developing dorsal brain. Although neither immunostaining nor in situ hybridization allowed to detect SHH in this compartment, more sensitive RT-PCR analyses indicate that SHH is present in the neocortex since E14.5 [52]. This possibility is compatible with our data showing the presence of the SHH receptor Ptch1 in the neocortex. The retarded diffusion of SHH into the perlecan-null cerebral wall could explain the delayed atrophy in this compartment in mutant mice. Nevertheless, additional direct evidence is still needed to conclusively establish that cerebral hypoplasia in the perlecan mutants is secondary to delayed SHH signal.

In addition to SHH, FGF signaling is required both for generating ventral precursors and for promoting their differentiation in the telencephalon, as nicely shown in the FGFR1; FGFR2 double mutant [53]. Mice lacking FGF2 exhibit a reduced density and number of neurons in the neocortex [54-56], and microinjection of FGF2 into the cerebral ventricles shortens the cell cycle and increases the number of cortical neurons [57]. In addition, the pallial defect of perlecan-null embryos strongly resembles that seen in mice with a conditional disruption of EXT1, the HS-polymerizing enzyme, which show an abnormally small cerebral cortex [58]. Therefore, less commitment to a neuronal fate in the perlecan knockouts could alternatively result from defective FGF2 signaling of the VZ progenitors in the cerebral wall. Admittedly, we did not detect overt changes in the intensity or pattern of distribution of FGF2 protein in the telencephalon of perlecan mutants. However, we note that growth factors such as FGF2 are required in very low amounts, undetectable by standard procedures, to activate signaling. Regulation of cell cycle and commitment to neuronal differentiation takes place before the last division of progenitors and during the following G_1 _[59]. Radial glia, as neural progenitor cells in the VZ [28,60-62], extend and retract basal processes to attach and detach from the basal lamina in a cell cycle-dependent sequence. In particular, VZ progenitors interact strongly with the basal lamina during G_1_, and perlecan-bound FGF2 could directly promote progression along the cell cycle and neuronal commitment at this stage.

## Conclusion

In summary, we show that perlecan influences the size of ventral and cortical telencephalic structures. Brain atrophy starts in the ventral telencephalon, with a marked reduction of basal progenitor proliferation, and progresses in a medial-to-lateral direction. Our findings suggest insufficient recruitment and/or signaling by the morphogen SHH, in the basal lamina of the floor plate. In addition, in the cortex the presence of perlecan in the leptomeningeal basal lamina could be crucial to provide signals to induce neurogenesis and cell cycle progression in the VZ progenitor population, either through direct contacts with radial glia processes or due to its ability to stimulate FGF2 interaction with cell receptors. Crosstalk between different signaling molecules and their cell receptors is necessary to maintain cell differentiation and several extracellular matrix proteins are known to facilitate these interactions. In this context, it is important to stress that perlecan represents an important suppressor of vascular smooth muscle cell proliferation [63] and of endothelial cell migration [64]. Finally, we note that our findings may have important implications to understand the pathology of several human conditions, such as holoprosencephaly [65], neuronal heterotopias [66] or microcephaly. Elucidation of the molecular mechanisms controlling proliferation and differentiation of specific subsets of progenitor cells in brain may lead to the development of strategies for neural stem cell production and its utilization in brain therapies.

## Methods

### Mice

Perlecan gene was inactivated in mice by homologous recombination in embryonic stem cells. As previously demonstrated [4], deletion of the sixth exon of the perlecan gene results in the lack of expression of perlecan protein. PCR was used to genotype offspring resulting from perlecan heterozygous matings. We used two sets of oligonucleotides for genotyping. The first set of primers, 5'-AACCAGAAGGGGTGGCAAGAA-3' and 5'-GCAGCACCTCTTGAATCTGAG-3', amplifies a fragment of 500 bp between intron 5 and exon 6, which is absent in the perlecan mutants. The second set of primers, 5'-AACCAGAAGGGGTGGCAAGAA-3' and 5'-TACTGAGGCAGAGTCTCTCTC-3', amplifies an approx. 1000 bp fragment between the intronic regions 5 and 6 in the wild-type allele. In the perlecan mutant allele this fragment is reduced to 500 bp. We used the same PCR conditions for both sets of primers: denaturation at 94°C for 3 minutes, followed by 35 cycles at 94°C for 30 seconds, 60°C for 30 seconds, and 72°C for 45 seconds, and a final step at 72°C for 3 minutes. Mice heterozygous for the mutation appeared normal and did not display any overt anatomical or behavioral abnormalities. Brains were removed and either fixed in Carnoy (60% ethanol, 30% chloroform, 10% acetic acid) or in 4% phosphate-buffered paraformaldehyde (PFA), embedded in paraffin, and sectioned at 6–7 μm. Sections were Nissl stained with cresyl violet.

All experimental procedures were in accordance with the Spanish and European Union legislations, and were approved by our Institutional Animal Care and Use Committees.

### Immunohistochemistry

Primary antibodies used were: rabbit polyclonals: anti-Sonic Hedgehog (1:50; Santa Cruz Biotech.), anti-Ki67 (1:50; Abcam, UK), anti-FGF2 (1:500; Santa Cruz Biotech.), anti-Perlecan domain II (1:5,000; [67]), anti-phospho-histone H3 (ser 10) (1:100; Upstate), anti-laminin-1 (1:100; Abcam, UK), anti-calbindin (1:5000; Swant); goat polyclonal anti-Patched (1:50; Santa Cruz Biotech.); monoclonals: anti-nestin Rat401 (1:4; Developmental Studies Hybridoma Bank, Iowa), anti-PCNA (1:100; Santa Cruz Biotech.), anti-Tbr1 (1:1000; R. Hevner, Seattle, USA) and anti-BrdU (1:2500; Vector Laboratories, UK). Fluorescent secondary antibodies included goat anti-rabbit IgG tetramethylrhodamine isothiocyanate (1:100; Abcam, UK) and goat anti-mouse IgG Alexa488 and goat anti-rat IgG Alexa488 (1:200; Molecular Probes). For non-fluorescent immunohistochemistry we used the following secondary antibodies: biotinylated anti-mouse IgG (1:200; Vector Laboratories), biotinylated anti-goat IgG (1:200; Vector Laboratories), and biotinylated anti-rabbit IgG (1:200; Vector Laboratories). Immunostaining was visualized by using the ABC Elite kit (Vector Laboratories) and a solution of 3,3'-diaminobenzidine (Sigma) and hydrogen peroxide. Images were obtained in a Zeiss Axioplan microscope equipped with an Axiocam camera or with a Leica TCS SP confocal microscope equipped with an Ar 488 laser and HeNe 543 and 568 laser lines. Confocal analysis was performed in the SCIE, University of Valencia and in the Instituto de Neurociencias de Alicante.

### BrdU experiments and apoptosis labeling and detection

To track proliferating cells, we injected dams intraperitoneally with 5-bromo-2'-deoxyuridine (Sigma) at 40 mg/kg of body weight. Embryos were collected at varying time points (30 minutes, 2, 4 or 24 hours) after injection. After a 30 min survival, BrdU labels cycling cells during S-phase. Two hours survival marks cells until M-phase, and four and 24 h survival allows the detection of S-phase cells and some postmitotic neurons. The endpoints of migration of early neuronal cohorts were similarly studied, with BrdU injections to dams at E12.5 and embryo collection at E17.5. BrdU staining was performed on paraffin sections that had been pretreated to denaturate DNA (2 N HCl for 30 min at 40°C and then neutralized in 0.1 M sodium tetraborate). Apoptosis was analyzed on paraffin sections of 4% PFA fixed brains by the terminal deoxynucleotidyl transferase (TdT)-mediated dUTP nick end labeling (TUNEL) method using a commercial in situ cell death detection kit (Roche) according to the manufacturer's instructions.

### Quantitative studies of regional growth of the telencephalon and of cell proliferation

To quantify brain size at E12.5–13.5, we measured the cross-sectional area of each telencephalic region considered on two sets of three coronal sections for each embryo at levels similar to those shown in Fig. 1B and 1E.

PCNA and Ki67 antibodies mark the same cells, namely the progenitor population [25,26]. In the present study, we initially immunostained such a population with a mouse monoclonal antibody to PCNA. However, in some experiments we used Ki67 antibodies instead of anti-PCNA so that double-label immunofluorescence could be performed along with anti-BrdU. We performed simultaneous immunofluorescence staining of Ki67 and BrdU after survivals of 30 minutes, 4 hours or 24 hours post-BrdU injection. We calculated the labeling index as the number of BrdU^+ ^cells divided by the number of Ki67^+ ^cells in a given telencephalic region. Quitting fraction was calculated in E12.5 embryos pulsed with BrdU and retrieved 24 h later. The quitting fraction represents the ratio of BrdU^+^, Ki67^- ^cells (i.e., those that have left the mitotic cycle) referred to the total number of BrdU^+ ^cells. Mitotic index was calculated as the number of phospho-histone H3^+ ^cells (cells in M phase) divided by the number of Ki67^+ ^cells in a given telencephalic region. We counted phospho-histone H3^+ ^cells in sections adjacent to those where we counted Ki67^+ ^cells. To compare the extent of G_2_/M phase between wild-type and perlecan-null E16.5 neocortex, pregnant mother were injected with BrdU 2 h before killing and double labeling was performed to reveal BrdU-positive nuclei as well as mitotic cells containing phospho-histone H3, as has been described [24]. We counted six areas in three sections for each region of the analyzed brains. On each litter, we compared mutant values with the mean value of two wild-type embryos. Statistical significance was analyzed using *Student'*s *t *test.

## Abbreviations

BrdU, 5-bromo-2'-deoxyuridine; CB, calbindin; CGE, caudal ganglionic eminence; ECM, extracellular matrix; FGF, fibroblast growth factor; HS, heparan sulfate; LGE, lateral ganglionic eminence; MGE, medial ganglionic eminence; PLN, perlecan; SHH, Sonic hedgehog; SVZ, subventricular zone; VZ, ventricular zone.

## Authors' contributions

AG collected brains, carried out immunoassays and confocal analysis, and performed quantitative studies and the statistical analysis. JM and CGS carried out immunoassays and histological analysis. AF participated in the design of the study and collaborated in writing the manuscript. MC conceived the study, participated in its design and coordination and wrote the manuscript. All authors read and approved the final manuscript.
